# ‘It'd Be Nice to Know if You're About to Have Drain Cleaner’: A Qualitative Study of Preferred Drug Checking Service Features to Inform the Design of CheQpoint, Queensland's First Fixed‐Site Drug Checking Service

**DOI:** 10.1111/dar.70003

**Published:** 2025-06-30

**Authors:** Cheneal Puljević, Nina Pocuca, Brodie C. Dakin, Monica J. Barratt, Cameron Francis, Daniel Stjepanović, Sara Gill, Geoff Davey, Leanne Hides

**Affiliations:** ^1^ Centre of Research Excellence on Achieving the Tobacco Endgame, School of Public Health, The University of Queensland Brisbane Australia; ^2^ The Loop Australia Australia; ^3^ National Centre for Youth Substance Use Research (NCYSUR), School of Psychology, Faculty of Health and Behavioural Sciences, The University of Queensland Brisbane Australia; ^4^ Criminology and Justice Studies, Social Equity Research Centre and Digital Ethnography Research Centre, RMIT University Melbourne Australia; ^5^ National Drug and Alcohol Research Centre, University of New South Wales Sydney Sydney Australia; ^6^ Queensland Injectors Health Network (QuIHN) Brisbane Australia; ^7^ Lives Lived Well Brisbane Australia

**Keywords:** drug checking, harm reduction, qualitative

## Abstract

**Introduction:**

Drug checking is an important harm reduction service that allows service users to make an informed decision about drug use. In early 2024, Queensland became the second Australian jurisdiction to implement a fixed‐site drug checking service (CheQpoint). To prepare for this new service, we conducted a qualitative study to explore perceptions of drug checking services and ideal service features among a sample of potential service users, including those experiencing substance dependence.

**Methods:**

Semi‐structured interviews were conducted with 57 adults (57% male) who had used unregulated drugs in the past 12 months, recruited via online advertisements and from Queensland‐based alcohol and other drug treatment services or a needle and syringe programme. Interviews focused on barriers and facilitators to accessing drug checking and opinions on service features. Data were analysed using iterative categorisation.

**Results:**

Six themes were identified: participants are highly motivated to use drug checking; barriers to access include confidentiality concerns, cost, wait time and accessibility; people will employ more harm reduction strategies following an unexpected test result; people experiencing substance dependence may be less motivated to access drug checking services and face greater barriers to service access; drug checking will impact the unregulated drug market and practices of people who sell drugs; services should be safe and comfortable spaces.

**Discussion and Conclusions:**

These findings highlight the importance of drug checking services like CheQpoint being confidential, free, conveniently located, welcoming and non‐stigmatising. Findings can be used to inform the implementation of future drug checking services to enhance their acceptability among service users.

## 1 Introduction

Within the context of drug prohibition, unregulated drugs can pose health risks through the presence of unwanted and unexpected compounds (e.g., fentanyl), or due to being stronger than expected [1, 2, 3]. As such, drug checking services have been introduced in approximately 26 countries [4], with Australia's first fixed‐site drug checking services implemented in the Australian Capital Territory and Queensland in 2022 and 2024, respectively [5, 6], while Victoria's festival‐based services have commenced and their fixed site is due to open in mid‐2025 [7]. Drug checking involves the chemical analysis of substances of concern; substances are submitted by members of the public, and results are returned to service users through a tailored conversation that aims to reduce drug‐related harms [1]. As such, drug checking services are an important source of harm reduction, allowing service users to make an informed decision about the drugs they intend to use, and to have a conversation with a trained professional about their drug use [1, 2, 3]. A secondary benefit of these services is their ability to generate timely information about drug markets; for example, public warnings about adulterated substances identified through drug checking can be disseminated rapidly via text message or social media alerts [8, 9, 10, 11].

There is a growing evidence base for the effectiveness of these services in reducing drug‐related harms [1, 2, 3, 12]. For example, most drug checking service users dispose of substances found to contain potentially dangerous adulterations [11, 12, 13, 14], use a smaller quantity when the substance is identified as being of high purity [2, 12] and/or alert their friends to substances of concern [12]. Despite these known benefits [1, 2, 3] and a high proportion of people reporting that they intend to use drug checking services (e.g., between 82% [15] and 94% [16] of Australian festival attendees), uptake of drug checking services among people who use drugs has been relatively low in some settings (e.g., some supervised injecting facilities in Canada [17, 18]). However, other services in Canada and the Netherlands have demonstrated relatively high levels of service engagement [19, 20], suggesting that uptake is not uniform across all contexts. This highlights the need for research to examine factors that may act as barriers and facilitators to accessing drug checking services among people who use drugs.

A few international studies have explored barriers to using drug checking services [16, 21, 22, 23, 24, 25, 26, 27, 28, 29, 30]. For example, fear of arrest by police was the most commonly reported barrier to using a hypothetical drug checking service among two samples of Australians reporting previous illicit drug use and attending a festival/licensed venue [16, 23]. Other barriers described in these quantitative Australian studies include giving up half or more of a tablet, capsule or point (0.1 g of a powder/crystal) for testing, paying more than $20 per test [16], or friends or relatives finding out about their drug use [23]. Qualitative studies among Canadians who use drugs have highlighted that acceptable drug checking services should be free, fast, accurate, confidential, anonymous, conveniently located, free of police presence or influence, trauma‐informed (e.g., accommodates the needs of patients with a history of significant trauma [31]), tailored to the diverse needs of their priority populations, staffed by people who use or have used drugs, integrated with existing health services, and require only a small sample of a drug to be provided for testing [21, 22, 24, 26, 29]. A US‐based study conducted among a sample of 22 people who use drugs identified time constraints, a high level of trust in their supplier and a belief that they were not at risk from fentanyl, xylazine or other adulterants as barriers to drug checking [25]. A survey of 1113 Austrians who use drugs similarly identified a high level of trust in their supplier as the main reason for not using a drug checking service, followed by confidence in substances' quality and inconvenient service locations [30].

While some studies have explored barriers to using drug checking services among Australians who attend music festivals and/or licensed venues (e.g., [16]) or international samples of people who use drugs (e.g., [21, 22]), there is a lack of research examining these barriers, in particular studies that include those experiencing substance dependence and qualitative studies that allow for an in‐depth exploration of these barriers. Understanding these barriers among a broad range of people who use drugs, including people experiencing substance dependence, is crucial to facilitate the implementation of acceptable drug checking services and targeted awareness campaigns that address barriers that may prevent people from using these services. As such, ahead of the implementation of Australia's second (and Queensland's first) fixed‐site drug checking service, CheQpoint, in early 2024, this study aimed to understand perceptions of drug checking services and their features, including barriers and facilitators to accessing such a service, among a broad sample of Australian adults who use unregulated drugs, including those who experience substance dependence, and represent potential future clients of the service. These findings will be valuable to inform the implementation of future drug checking services in Australia and elsewhere, to enhance these services' acceptability among service users.

## 2 Methods

Participants who were 18 years and older and had used unregulated substances in the past 12 months were recruited to complete an online survey about perceptions of drug checking services. Survey respondents were then invited to complete a qualitative interview to elaborate on their survey responses. This manuscript reports the results of the qualitative interviews; survey findings will be published separately.

### 2.1 Participants and Recruitment

Survey participants were recruited either online via social media posts distributed by Queensland‐based alcohol or other drug (AOD) organisations, research groups, and a music festival supportive of drug checking, or via staff at two not‐for‐profit Australian AOD organisations providing health services to people who use drugs: Lives Lived Well (LLW) or Queensland Injectors Health Network (QuIHN). LLW participants were recruited from inpatient residential AOD treatment services in South‐East Queensland. QuIHN participants were recruited on‐site at QuIHN Brisbane while attending the service's needle and syringe programme. Other health services offered by QuIHN for people who use drugs include counselling, a general practice medical centre, peer‐supported Hepatitis C treatment, outreach services and support groups [32].

### 2.2 Procedure

Individuals interested in participation were provided with an online information sheet and asked to provide informed consent. Participants who consented then completed a 15‐min online survey. All QuIHN and LLW participants and the first 20 online participants who completed the online survey were invited to complete the interview. Semi‐structured interviews were conducted by one male co‐author employed as a research assistant (B.C.D.) and two female research assistants (M.C. and G.N., both female) who were completing postgraduate degrees in clinical psychology and had experience working with people experiencing substance dependence. None of the interviewers had prior relationships with any participants.

Interviews were conducted in‐person with participants recruited via LLW and QuIHN, and via Zoom with participants recruited online. Interview questions were developed by members of the research team based on existing literature (e.g., [16, 21, 22, 23]), and generally covered concerns about the contents or quality of unregulated drugs, and perceptions of drug checking services (with a focus on barriers and facilitators to using a service), service features and testing methods (see Appendix 1 for the interview protocol). The average interview duration was 34 min. Participants who completed the interview were paid $40 AUD as a reciprocity payment. Interviews were audio recorded with participant permission and transcribed by a research team member using Otter, or by a professional transcription service.

### 2.3 Data Analysis

NVivo (1.7) software was used to analyse data using iterative categorisation [33] using a combined deductive (using the interview schedule and authors' familiarity with literature) and inductive (e.g., theme 5) approach. First, B.C.D. coded all interviews and created a qualitative codebook. S.G. then used the codebook to independently code six interviews (10%) to ensure consistency. Intercoder reliability was then calculated by N.P., with discrepancies between the two coders (i.e., Cohen's Kappa < 0.41) reviewed and resolved by N.P. B.C.D. then coded the major themes and sub‐themes arising from the coded data. This study followed the consolidated criteria for reporting qualitative studies (COREQ) checklist (see Table S1) [34].

### 2.4 Ethical Approval

The study received ethical approval from The University of Queensland's Human Research Ethics Committee (2022/HE002006).

## 3 Results

### 3.1 Participants

Of 118 participants that completed the online survey, 57 (47%) completed a qualitative interview (18 LLW; 20 QuIHN, 18 Online). One participant did not complete the online survey due to self‐reported limited literacy but did complete the qualitative interview.

Our study sample includes a diverse group of people who use various drugs (see Table 1). Most participants were male (57.1%), with a smaller proportion identifying as female (37.5%) or non‐binary (1.8%). Most participants identified as heterosexual (71.4%), while others identified as bisexual (16.1%) or homosexual (8.9%). Educational completion ranged from early high school (33.9%) to undergraduate (21.4%) or postgraduate (3.6%) degrees. Similar proportions reported never injecting drugs (39.3%), injecting drugs in the past 3 months (41.1%), or injecting drugs but not in the past 3 months (19.6%). In the past 3 months, amphetamines (78%) and cannabis (72%) were the most commonly used drugs, with substantial proportions also reporting use of opioids (63%), MDMA (42%), hallucinogens (42%), and/or cocaine (33%), demonstrating the sample's diverse drug use patterns (i.e., including people who predominantly use ‘party drugs’ like MDMA and those who inject opioids).

**TABLE 1:** Interview participants' socio‐demographic characteristics and history of injecting drug use.

Characteristic	*N* (%) (*N* = 56)
Gender	
Male	32 (57.1)
Female	21 (37.5)
Non‐binary	1 (1.8)
Transgender	1 (1.8)
Other	1 (1.8)
Median age (interquartile range, range)	33.5 (16, 18–54)
Sexuality	
Heterosexual	40 (71.4)
Homosexual	5 (8.9)
Bisexual	9 (16.1)
Prefer not to answer	2 (3.6)
Aboriginal and/or Torres Strait Islander	13 (23.2)
Education	
Completed up to Grade 10	19 (33.9)
Completed up to Grade 12	12 (21.4)
Completed a diploma/trade certificate	11 (19.6)
Completed an undergraduate degree	12 (21.4)
Completed a postgraduate degree	2 (3.6)
History of injecting drug use	
Never	22 (39.3)
Yes, in the past 3 months	23 (41.1)
Yes, not in the past 3 months	11 (19.6)
Moderate‐to‐high risk substance use (*n* = use in past 3 months)^a^	
Cannabis (*n* = 50)	36 (72%)
Cocaine (*n* = 42)	14 (33%)
Amphetamines (*n* = 45)	35 (78%)
MDMA (*n* = 43)	18 (42%)
Sedatives (*n* = 42)	29 (69%)
Hallucinogens (*n* = 40)	17 (43%)
Opioids (*n* = 35)	22 (63%)

^a^ Among those who used the substance in the past 3 months, a score of > 3 on the World Health Organization's Alcohol, Smoking and Substance Involvement Screening Test (WHO ASSIST). The number of participants who reported using each of the listed substances in the past 3 months is noted in brackets. A score of > 3 on the WHO ASSIST is indicative of moderate‐to‐high risk use for the listed substances, which indicates the likelihood of dependence and health, social, financial, legal or relationship problems.

### 3.2 Interview Findings

Six themes were derived from the interviews. Quotes supporting each theme are included below, with additional quotes for each theme presented in Table 2.

**TABLE 2:** Further examples of quotes for each theme.

Theme	Example quotes
Theme 1: Participants are highly motivated to use drug checking to understand the contents of their drugs	‘Like if people did have the opportunity to actually test to see if there was something bad in it, then they wouldn't take it. Like I think people are pretty smart in that, like, if they were given the information, they would, yeah, they wouldn't take it… I don't necessarily think it would stop people from taking drugs in general. But I think that it would help make people make better decisions and like safer decisions with drugs’. (Male, 25 years, online participant) ‘I think it [drug checking] is needed. It's valuable as a harm minimisation tactic for the whole community and especially at festivals and things. I see where they're coming at with that, because there is typically and probably a big scope of people who aren't experienced in whatever they're partaking in, so it's a good way to minimise the risks’. (Male, 43 years, LLW participant) ‘I think it will protect those that are beginning to experiment the most… So, I think it's a good way to reduce harm within the community and have a safer reliable way to take substances without, you know, having a bad time or something going wrong… I think it's definitely like a safety net for those that do use substances, from going down to further, you know, health problems and whatnot’. (Male, 31 years, online participant)
Theme 2: Barriers to access include confidentiality concerns, cost, wait time, and accessibility	‘Police intervention would be my biggest barrier. The risk of being, I guess, punished or apprehended or some sort of legal repercussions’. (Male, 39 years, online participant) ‘If there was too great of a cost for testing, that could create a massive barrier. It should be free to the public, or next to free, if that makes sense’. (Male, 36 years, QuIHN participant) ‘Probably the time [is the biggest barrier]. I think if it took too long. It took any more than like an hour or something to get back the results then it's probably, a lot of people would say, including me, just like “ah that's a mess around doing that”’. (Gender not specified, 23 years, LLW participant) ‘I think that's going to be a big barrier if [the drug checking service] is situated in a separate place and people—like drug users are having to go to a place they wouldn't normally go to just for that’. (35 years, online participant)
Theme 3: People will employ more harm minimisation strategies following an unexpected drug checking test result	‘Harm reduction … Say, for example, two tablets of that is equivalent to this or this, just so you know that. Because you might think it's shit and take copious amounts, but it could be really fatal. So it'd be good to get information about how that drug will react in our body’. (Male, 26 years, LLW participant) ‘I suppose I'd want more information on what it actually is and what that harm reduction effect could look like, like harm reduction information about it. What that could look like in a poly substance setting and I would likely consume the drug if there was not any, like immediate instant danger issues, like say, its not fentanyl, or something like that in the substance. And, yeah, be more mindful. Like if it was something that wasn't extremely harmful, that I could still use, but is warned not to consume alcohol with or not to mix with another substance, I would, I would definitely still consider taking a substance’. (Female, 27 years, online participant)
Theme 4: People experiencing substance dependence may be less motivated to access drug checking services and also face greater barriers	‘I think with people who are reliant on drugs to survive they're going to need to know that it's a really high risk before they would not take the drug. … If a test is just saying that this dangerous substance is detected—yeah, I don't think that's going to be convincing for a lot of people to go out of—get out of harm's way with taking it—you know? They're just going to be like well, maybe it's like 2% of the dangerous substance in there so I'm just going to take it’. (Gender not specified, 35 years, online participant) ‘I think the main barrier [to accessing drug checking] would be just when you're—when you're in addiction your priority is just to get high. So, if you get high, you've accomplished what you want. I think that would be probably the main barrier, that it [drug checking] just wouldn't be a priority’. (Female, 32 years, LLW participant) “As an ex‐heroin user, like there was no way I'd be parting with any of my heroin that I worked really hard to get to be tested. Like I would still have used it anyway.” (Female, 40 years, QuIHN participant)
Theme 5: Drug checking will impact the unregulated drug market and how people who sell drugs operate	‘I would say most dealers want to make sure their drugs are safe, because that's how they get additional customers, right? No one's going to like, if you get some bad stuff from someone, you're not going back’. (Male, 25 years, online participant) ‘When I was selling drugs it would have been good to know how pure the drugs I was selling were. Yeah, definitely would have helped out a lot in a lot of situations to know what was in them. And how pure it was. Or unpure it was’. (Male, 26 years, LLW participant) [if test showed methamphetamine is not present in drug sample] ‘I'd go back to my dealer to be honest and be like “what the fuck is this?” Yeah, and show them the test… that's honestly what I'd do’. (Male, 33 years, LLW participant)
Theme 6: Drug checking services need to be safe and comfortable spaces	‘I think that people with lived experience is very different than someone that just read from a book and is practicing. So, if it was someone like a doctor, or someone without lived experience, I wouldn't want to really know about it, and I wouldn't want to talk to them’. (Female, 32 years, LLW participant) ‘I'd love for it to be peer led and proud of that, at all levels of the service. Peer led and in the places it needs to be, so at every festival, at the clubs. I think that's how you would market it, make it in the spaces that people are consuming substances’. (Male, 36 years, QuIHN participant) ‘I really stress the importance of ensuring that health care professionals are really educated in that reduction of stigma. And yeah, hopefully are quite socially and emotionally intelligent in their approach with people, and understanding that it's probably like until this gets, I guess, more normalised in Australia, like it's going to be a bit nerve wracking for people to engage’. (Female, 27 years, online participant)

### 3.3 Theme 1: High Motivation to Use Drug Checking Services to Understand Drug Contents

Participants were overwhelmingly in support of drug checking, with most reporting strong personal motivation to access such a service.

‘Yes I would use it. And I would shout it from the rooftops.’ (Female, 47 years, online participant)

‘I think it's better to be able to know what you're taking than not know. It'd be nice to know if you're about to have drain cleaner.’ (Male, 37 years, LLW participant)

Motivations for accessing drug checking centred on the desire to understand the drug contents, particularly the presence of adulterants. Many participants commented on the increased need for drug checking in Australia due to their perceptions of the diminished quality of unregulated drugs within Australia over recent years, and awareness of the presence of fentanyl in overseas drug markets.

‘I've definitely noticed more issues with drugs being cut and purity decreasing. Yeah, it's a bit scary out there at times … Yeah, you know, like even being at festivals, you're hearing constantly about someone taking a substance and having a reaction to it that probably shouldn't be associated …. I think having the capacity to test would be a really powerful way to protect our community.’ (Female, 27 years, online participant)

### 3.4 Theme 2: Barriers to Access Include Confidentiality Concerns, Cost, Wait Time, and Accessibility

Although all participants displayed enthusiasm for drug checking, they also mentioned several barriers that would either prevent or discourage them or others from accessing drug checking. The four most emphasised barriers were confidentiality (and resulting legal concerns), cost (of giving up drugs for testing or of using the service), wait time and accessibility issues.

Confidentiality was the most commonly mentioned barrier, with participants raising concerns that police would target people accessing drug checking services, given that they are in possession of unregulated drugs. Participants' confidentiality concerns extended beyond fear of police involvement to concerns that family members, work colleagues, or other members of society may see or hear of them accessing drug checking, and thus assume that they are someone who uses drugs.

‘Well police intervention and police surveillance, being recognised, being spotted … I'm sure that wouldn't go down very well at all, and it could be a family member, it could be anybody just so I wouldn't like to be identified at all.’ (Male, 51 years, QuIHN participant)

‘The fear of being reprimanded for possession or, you know, walking in with it and then leaving and then being arrested for possession of an illicit substance. That would be the biggest deterrent. Yeah. Criminal charges.’ (Male, 37 years, LLW participant)

The second most emphasised barrier to drug checking was cost—either related to giving up drugs for testing, or the monetary cost of using a service. Participants' aversion to relinquishing a part of their drug for analysis was dependent on the quantity that people would have to give up for drug checking (e.g., 1 point vs. 1/10 of a point). However, some participants commented that giving up *any* drugs (even 1/10 of a point) was a barrier to using the service. Participants were also hesitant to pay money for drug checking; some highlighted that this may only be a barrier if the cost is significant (e.g., greater than $10 per test), while others commented that the tests would have to be free for them to consider using the service.

‘Costs would definitely be number one [barrier], because I guess you've got to think about, how much am I spending on the drugs? Like, how much of the drug am I losing to get a sample done?’ (Female, 21 years, online participant)

Third, participants remarked that the length of time that service users would have to wait for test results may deter people from accessing the service, particularly if wait times were longer than 15 min. This was driven by factors such as impatience and desire to use substances as soon as possible, and related discomfort at having to wait for results due to confidentiality concerns and fear of potential legal repercussions from using the service.

‘I think if it took too long … If it took any more than like an hour or something to get back the results then it's probably, a lot of people would say, including me, just like “ah that's a mess around doing that”.’ (23 years, LLW participant)

The fourth most commonly mentioned barrier was accessibility. Participants described the general inconvenience of having to travel to a location to have drugs tested as a potential deterrent. This was particularly seen as a barrier if the service could not be accessed via public transport or was located somewhere that people who use drugs do not usually frequent. As such, participants recommended that such a service would benefit from being co‐located with a service that people who use drugs already attend. This would allow familiarity with the setting and staff, and offset much of the aversion and fear associated with entering a drug checking service.

‘People might be more likely to be willing to engage with the service in that way if it was through a needle and syringe program, or somewhere they already existed, or somewhere where they already attended, you know?’ (Female, 46 years, QuIHN participant)

### 3.5 Theme 3: Greater Barriers and Lower Motivation Among People Experiencing Substance Dependence

Several participants also highlighted how the service would not be as readily accessed by people currently experiencing substance dependence, compared to, for example, people who use drugs occasionally (e.g., at music festivals). For example, participants perceived that people experiencing substance dependence may be less willing to relinquish a sample of their drugs for drug checking, given associated costs.

‘Yeah, and then a 10th of a point, like, that seems a lot … when you've kind of had to rake up money all day to get up the money to use, you know, 10% of what you've just bought does seem a lot like it is a lot … And, yeah, it can be hard to access and not as available if you would like it to be sometimes. So, having to even give up that 10% could hurt.’ (Female, 46 years, QuIHN participant)

Second, while wait time was described as a barrier to using drug checking services among most participants, people experiencing substance dependence may be even less inclined to use drug checking if there is any wait time involved with the results, given the strong urgency to use drugs stemming from cravings or withdrawal symptoms.

‘Yeah, I mean when I've got drugs, I'm just straight into that bag or straight into that pill. You know, I'm not gonna hang around and wait for it to get tested.’ (Male, 33 years, LLW participant)

Participants also discussed how the relationship between people experiencing substance dependence and the police and legal system is a further barrier to using a service. Participants described how people experiencing substance dependence have usually had negative experiences with police, and that their distrust of police and the legal system may make them more paranoid and disinclined to access drug checking, due to fear of police involvement.

‘In the drug world, people don't like the police. They see them as people that take their drugs and send them to jail for things that they shouldn't be getting sent to jail for … all drug addicts are paranoid, so they'd probably be paranoid that there's going to be an arrest or a search after it [accessing drug checking].’ (Female, 29 years, LLW participant)

### 3.6 Theme 4: Unexpected Test Results Motivate Engagement With Harm Reduction Strategies

Overall, there was a high level of interest in receiving harm reduction information when accessing a drug checking service, specifically information on potentially dangerous drug combinations, dose‐to‐body weight ratio and what to do if experiencing adverse symptoms. Participants indicated that receiving this information would increase the likelihood that harm reduction is practised when using unregulated drugs (particularly for people with less drug use experience).

‘… information about the risks associated with the drug they're taking, and how they can potentially mitigate those risks … what to do if something does go wrong, because it does happen and a lot of people aren't prepared for that … people aren't going to go out and seek out that information themselves a lot of the time … So, I feel like that this is probably the best place to get reliable information.’ (Female, 21 years, online participant)

Participants also mentioned that being informed of the presence of potentially hazardous adulterants or that their drugs are high purity would change *how* they consumed the substance; while receiving these unexpected results may not deter all people from using the tested substance, these participants indicated they would employ greater harm reduction behaviours in these situations. This might include consuming less than originally intended, sampling a very small amount first and monitoring effects, or making sure they were around other people when they consumed the drug. Finally, participants discussed that if a drug checking result indicated adulteration with fentanyl, they would ensure naloxone was on hand to reverse potential overdoses.

‘[If a novel psychoactive substance was identified], I guess I would potentially still use it but I'd be a lot more careful in how I was using it and probably make sure I was with someone that I trusted … So I would change my behaviour around the drug.’ (Gender not specified, 35 years, online participant)

‘[If fentanyl was detected], I would proceed with caution. Yeah, I would have a hell of a lot less. And yeah, I could just make sure that I use with someone else and that we had Naloxone available. And that I've communicated it to someone.’ (Female, 46 years, QuIHN participant)

### 3.7 Theme 5: Drug Checking has the Potential to Impact the Practices of People Who Sell Drugs

A few participants mentioned that drug checking has the potential to impact the unregulated drug market as people who sell drugs will be motivated to use the service, thus incentivising the sale of drugs that contain only the advertised substance.

‘I imagine you only need to test a certain proportion of the market to have a significant influence on decreasing the amount of adulteration that would happen, which is what you see in Europe. They have very high quality and high purity MDMA because so many people test their stuff, it's like well there's not much of a market for adulterated substances.’(Male, 41 years, online participant)

Participants discussed several different motivations that people who sell drugs might have to access drug checking services, such as wanting to inform their customers of the contents of the drugs or only sell products which do not contain unexpected adulterants or are not very high purity, to decrease the likelihood of customer attrition (e.g., due to dissatisfaction, health hazards, or death via overdose or poisoning). Another motivation is that they can then cut higher purity products to increase the quantity of drugs they can sell and maximise profits.

‘When I was selling drugs it would have been good to know how pure the drugs I was selling were. Yeah, definitely would have helped out a lot in a lot of situations to know what was in them.’ (Male, 26 years, LLW participant)

‘Probably I would use drug checking if I was dealing … Yeah, like to make sure people are getting what they are buying and when you're dealing people want to know who's got the better stuff, it's a competition.’ (Male, 22 years, LLW participant)

Participants also commented on how the existence of accessible drug checking stands to make people who sell drugs more accountable. For example, people who buy and use drugs may expect suppliers to provide detailed information on the purity and contents of the drugs they are selling or test the product that they have purchased to identify poor quality substances. Several participants commented that receiving unfavourable results would motivate them to either confront, report, or no longer purchase drugs from the person who sold them the drug. These participants predicted that some people who sell drugs may thus dislike drug checking and their customers accessing this service due to potential negative implications for their business.

‘It makes people accountable; do you know what I mean? … It will flush out all the dickheads out there that are selling drugs to people that really shouldn't be selling drugs … They're not smart enough, you know, for a start and … they don't give a fuck about the person, they just want the money.’ (Male, 49 years, QuIHN participant)

### 3.8 Theme 6: Drug Checking Services Need to Be Safe and Comfortable Spaces

Participants described how the nature of the staff, the ‘culture’, and the staff‐client interactions at a drug checking service impacts service uptake. Several participants highlighted that the process of entering a drug checking service may be uncomfortable and anxiety‐provoking for many. This may arise simply from the nature of entering a service while carrying unregulated drugs, and having to hand over drugs and have a discussion with staff about drug consumption, a behaviour that is usually done with marked discretion. As such, people accessing drug checking services will likely be in an uncomfortable and vulnerable state, and thus staff interactions would be crucial for determining whether they would return or recommend the service to others.

‘It's intense for a drug user … Because drug users are normally shunned, and in society looked down upon … It's kind of like stepping out of your comfort zone really.’ (Male, 38 years, QuIHN participant)

‘The attitude of staff [may be a barrier to access]. I don't want anyone looking down their nose at me, and that's what you commonly find.’ (Male, 49 years, QuIHN participant)

Many participants emphasised the importance of the service being ‘peer‐led’ or at least staffed with peers (i.e., people who use drugs) in client‐facing roles. Peers were perceived as less judgmental and preferable to interact with compared to health professionals, who were often perceived as more judgmental and less able to understand or connect with people who use drugs. Other participants expressed an ideal vision of drug checking services having both peers and health professionals collaborating to provide a service that is professional and knowledgeable but also warm and welcoming to clientele.

‘I guess just peer workers and health workers working together might reduce some of the barriers or intimidation. Not just for me, but, for probably some of my mates as well, you know?’ (Female, 46 years, QuIHN participant)

Participants also commented on how the service's ‘space’ will shape client engagement; these participants expressed the necessity of the service being ‘non‐clinical’, and having an atmosphere that is more casual than clinical.

‘I think that connection to the community is really important. And it not being too much of a clinical space, you know, to be I guess comfortable and welcoming and warm, not just kind of a clinical service, you know?’ (Female, 46 years, QuIHN participant)

## 4 Discussion

This qualitative study found a high level of motivation to use drug checking services among a sample of 57 Australians who use unregulated drugs, as service use would facilitate informed decision making about drug use and provide access to harm reduction advice. Participants also described several factors that may facilitate or hinder their likelihood of using drug checking services. Understanding these factors is crucial to the design and implementation of drug checking services, such as CheQpoint, so that they are considered acceptable to the target population. This information could also facilitate the development of targeted communication strategies to address potential barriers to service use, thus increasing the likelihood that people who use drugs will use these important services to reduce the likelihood of experiencing drug‐related harms.

The most commonly mentioned barrier to accessing drug checking services was confidentiality, specifically fear of potential legal concerns should police be present in or near the service or that people they know may see them using the service, and thus know that they use drugs. This barrier is also commonly described in previous international studies exploring barriers to using drug checking services [16, 21, 22, 23]. Fortunately, in jurisdictions where drug checking is operational (Australian Capital Territory and Victoria), police services have an operational protocol [35, 36] or in the case of Victoria, legislation has been passed, which specifies that police are not to conduct unnecessary person checks in the vicinity of harm reduction services. A 2022 review of factors impacting engagement in community‐based drug checking services by Masterton and colleagues identified explicit agreements with supportive police forces and a supportive police culture relating to harm reduction as key facilitators of drug checking service implementation [37]. Furthermore, policy guidelines for these Australian jurisdictions stipulate that drug checking services should be free and confidential [38, 39]. For example, at CheQpoint, clients could use the service anonymously as no identifying information was requested, and while clients were asked to provide demographic information (e.g., sex, age and postcode), this is optional. Participants' fear of their unregulated drug use being discovered also highlights the stigma that people who use drugs continue to experience, with this stigma a commonly identified barrier to using drug checking and other services designed for people who use drugs in international literature [22, 28, 31]. As such, it is essential that drug checking services are trauma‐informed [22, 31], and are not inadvertently reinforcing experiences of stigma that are a common experience for people who use drugs [22].

On a related note, various participants also described how the process of entering a drug service may be uncomfortable and anxiety‐provoking for many, likely resulting from previous stigmatising experiences when accessing health care or other services, as described in previous studies conducted with people who use drugs [21, 22, 23]. Participants thus reinforced the benefits of the service being welcoming, non‐clinical and casual, and ideally co‐located with an existing service designed for people who use drugs. It is for these reasons that CheQpoint was co‐located within QuIHN, and QuIHN staff have put notable effort into making their space as colourful and as welcoming to people who use drugs as possible. Participants also described the service being staffed by peers (i.e., people who use drugs) as a facilitator to using the service. In response, most of CheQpoint's healthcare workers were peers with lived or living experience of drug use, a recognised facilitator of health service access among this cohort [40, 41]. This finding also highlights the importance of ongoing quality monitoring to ensure that the services continue to be welcoming, professional and non‐stigmatising for everyone who uses them. The review by Masterton et al. similarly identified drug checking services being integrated into existing services with a core harm reduction ethos and being staffed by peers with lived experience of drug use as key facilitators of successful service implementation [37].

Other barriers to service use identified by participants included cost (of giving up drugs for testing or of using the service), wait time, and accessibility issues. Fortunately, CheQpoint was free, and service users were only asked to provide a very small amount (e.g., 1/10th of a point) for testing. Service wait times are (currently) minimal, with clients attending the service during its first 9 months of operation in April–December 2024 only waiting an average of 21 min for their sample to be analysed [42]. Finally, with accessibility issues in mind, both services were located in city centres, within a short walk to public transport, with accessibility described as a further enabler of successful drug checking service implementation in the review by Masterton et al. [37] and other international studies [21, 28, 29].

A handful of participants noted that service access may not be uniform across all population groups. For example, these participants perceived that people who experience substance dependence may be less likely to access the service compared to people who use drugs occasionally (e.g., at music festivals), due to the presence of strong urges to use drugs as soon as possible (e.g., to avoid withdrawal symptoms). While the drug checking service being co‐located with another service regularly accessed by people who use drugs may facilitate use of the services among some people who use drugs frequently [37], this finding points to a need for services to consider how best to serve these often hard‐to‐reach populations. One possible approach is a mobile drug checking clinic, such as the Drug Checking Van offered by the New Zealand Needle Exchange Programme [43], outreach workers who aim to reach those not engaged with relevant services [44], and/or targeted awareness campaigns (e.g., distributed on AOD organisations' social media) that explain the services' procedures, promotes their benefits and builds confidence about the service's confidentiality and lack of police presence.

Finally, several participants noted that drug checking services are likely to be used by people who sell drugs to monitor the purity and composition of the drugs they sell, and that there are benefits of this in incentivising the sale of ‘higher quality’ drugs. This finding is consistent with studies conducted in Canada that have found that people who sell drugs can play an essential role in reducing drug‐related harms and ‘empowering consumers’ [45] by sharing information about drug contents information with their clients [24, 45, 46, 47, 48]. For example, two studies conducted with people who sell drugs found that these participants reported concern about the safety of their customers [24, 47], with participants in one study describing using results from drug checking services to tailor their drugs, return dangerous batches and modify fentanyl to make it safer to consume [47]. As such, there are clear potential impacts of drug checking services beyond the individual level, especially as many people who buy drugs report a high level of trust for those who sell them drugs [48]. This form of ‘community‐wide’ [24] drug checking thus has the potential to benefit everyone who uses and sells drugs, from high‐level organised sellers to those who only sell to support their own use and/or provide drugs to friends [47]. A further example of a relevant ‘community‐wide’ drug checking initiative is the Vancouver‐based Drug User Liberation Front, which established an unsanctioned compassion club where members could purchase illicit drugs that had been analysed to ensure they were of known content and purity [49]. The compassion club was associated with reductions in all types of non‐fatal overdoses, highlighting the potential value of alternative models of drug checking [49].

### 4.1 Strengths and Limitations

While the Australian existing research in this space focuses on people who consume substances infrequently or at music festivals [23], a strength of the present study is the inclusion of a broad range of people who use drugs, including a substantial proportion of people with moderate‐to‐high severity of drug use (indicating likely dependence), history of injecting drugs and/or attending AOD treatment, which are often understudied populations. Another strength is the relatively large sample size in comparison to other qualitative interview research in this area [21]. Nonetheless, the present study presents the views of 57 people who use unregulated substances recruited from one geographical location (Queensland), and participants did not yet have experience with using drug checking services, meaning that our findings are limited to participants' perceptions of drug checking services rather than their reported experiences with or behaviours after using such services. Furthermore, we asked participants about their perceptions of drug checking services in general without discussing the specific equipment used or distinguishing between fixed site and festival‐based services. However, in Queensland the fixed‐site and festival‐based services used the same equipment (Fourier Transform Infrared Spectroscopy), procedures and health conversation. Given this uniformity, distinguishing between service types in our interviews was not deemed a priority. For the same reasons, we did not investigate differences in findings between people with different drug use patterns beyond indicating whether each quote is from an online participant (i.e., more likely to be a person who uses drugs at festivals) vs. a participant recruited from LLW or QuIHN (i.e., more likely to be dependent on substances), with our analysis instead focusing on overarching themes relevant to all participants. As such, we were also unable to explore how the ‘hierarchy of substance use’ (i.e., where using drugs like opioids may be perceived more negatively than using drugs like MDMA) may affect experiences of internalised stigma that prevent people from accessing services like drug checking services [26].

## 5 Conclusion

This qualitative study identified a high level of motivation to use drug checking services. Participants highlighted benefits of the services beyond identifying drug composition, including access to tailored harm reduction information and the potential positive impacts on the overall drug market that could result from people who sell drugs using the service. Potential barriers to service use include concerns about confidentiality, cost, wait time and accessibility, and participants noted that people experiencing substance dependence may be less likely to use the service due to wanting to use their drugs as soon as possible. These findings are useful for designing and implementing future drug checking services across Australia and elsewhere, to ensure that these services are considered acceptable by their users, and for designing tailored awareness campaigns that promote service features and benefits, thus enhancing uptake of these services recognised for their potential in reducing drug‐related harms.

## Author Contributions

**Cheneal Puljević:** conceptualisation, methodology, writing – original draft, writing – review and editing. **Nina Pocuca:** conceptualisation, formal analysis, methodology, project administration, supervision, validation, writing – original draft, writing – review and editing. **Brodie C. Dakin:** formal analysis, writing – original draft, writing – review and editing. **Monica J. Barratt:** writing – review and editing. **Daniel Stjepanović:** writing – review and editing. **Cameron Francis:** conceptualisation, methodology, writing – review and editing. **Leanne Hides:** conceptualisation, funding acquisition, methodology, supervision, writing – review and editing.

## Conflicts of Interest

C.F. is the paid CEO of The Loop Australia and C.P. and M.J.B. are unpaid volunteers. D.S. has collected data on behalf of The Loop Australia as an unpaid volunteer. The Loop Australia is a not‐for‐profit organisation that delivers drug checking services in Australia, and is one of the three partners operating Queensland's first fixed‐site drug checking service (CheQpoint), alongside QuIHN and the Queensland Injectors for Advocacy and Action (QuIVAA). The other authors declare no conflicts of interest.

## Supporting information


**Table S1.** Consolidated criteria for reporting qualitative studies (COREQ): 32‐item checklist.

## Data Availability

Data are available upon reasonable request.

## Appendix 1 Interview Guide

The following are example questions and structure for the interview portion of the study. Interview questions are designed to be used as a guide only and in a conversational manner, allowing for a discussion with the interviewee rather than a straightforward question and answer format. Open‐ended questions are used throughout to encourage the interviewee to express their views.

1. Are you worried about the contents, purity or quality of illegal drugs? Why/why not?
2. What do you think of the idea of a drug checking service?
3. How likely would you be to use a drug‐checking service if it were available? Why/why not?
4. In your opinion, what would be some of the barriers to accessing a drug checking service?
5. In your opinion, what would facilitate (make it easier) or make people more likely to access a drug checking service?
6. If you use a drug checking service and test results show that the drug you tested contained the substance you thought it did, what kind of information would you like to receive about that substance? (e.g., you thought you were testing methamphetamine and testing indicates the presence of methamphetamine).
  – For instance, would you like to receive information regarding how many people your age and sex use this drug? Or information regarding the health risks associated with using this drug?
7. If you use a drug checking service and test results show that the drug you had tested contained some other dangerous substance(s), what kind of information would you like to receive about that substance? (e.g., you thought you were testing methamphetamine and testing indicates the presence of another illicit substance or another substance cut/mixed with the methamphetamine)
  – How would you feel if the discovery of this dangerous substance in your drug sample was then shared with others via a ‘dangerous drug alert’? Note: No identifying information about you would be included in the alert, so no one would know that the alert came from your sample.
  – What do you think would be the best way to communicate this dangerous substance alert to others? (e.g., via print‐out posted on the wall at the drug checking service; online; SMS alert).
  – What would this alert look like (e.g., in terms of wording and length).
8. If you use a drug checking service, would you like the opportunity to talk to someone about your substance use?
  – If yes—how long would you want to spend talking to someone about your substance use?
9. What do you think of the idea of the drug checking service collecting some information about you such as your age, sex/gender, alcohol, and substance use history, current medications, in order to provide health advice that is more tailored towards you with the results of your drug checking test?
10. Have you ever tested the contents or purity of a drug? (e.g., on your own using a colour‐reagent kit; via a drug checking service (e.g., fixed site service or at a festival either in Australia or elsewhere); or via a mail‐in drug checking service).
  – No
  – Yes
    10.1 What method did you use?
    10.2 Did you receive any feedback on the results? If yes:
      - How did you receive the results of the test?
      - Did you find the process or the findings of the test interesting/not interesting?
      - Were you surprised by the results?/was anything unexpected?
    10.3 Overall, how did you find your previous drug checking experience/s?
    - Any aspects you liked/did not like about the experience/s?
    - Anything you found particularly helpful/unhelpful?
    - Anything you would change about/add to the experience/s?
    - Anything missing from the experience?
    10.4 What did you do with the drug after receiving the test results? (e.g., used the drug (or held onto the drug for future use); disposed of the drug; reported results to friends)
11. Drug checking (also known as pill testing) is a service that identifies the composition of drugs which are confidentially and voluntarily submitted by people who use drugs. Results of the tests are provided back to a service user by a health professional. The drug checking process looks something like this…
  You are asked to place a small amount of powder or part of a tablet/capsule (about 10 mg, which is one tenth of a point, as shown on the below image) into a clip seal bag, which is dropped into a locked box.
  You are asked to complete a short anonymous questionnaire asking the following questions:
  - What do you think the substance is?
  - Have you already tried some of this substance, and if so were the effects as expected/not as expected
  - Are you planning to inject this substance?
  Our chemist will then analyse the substance. This takes about 15–20 min. A health professional will have a brief chat with you to collect non‐identifiable information about you including your age, gender, cultural background, and employment status. This process also includes an assessment of your alcohol and other drug use, and your general health. The health professional will then provide you with the result of the chemical analysis and information on harm reduction strategies relevant to your individual needs. This takes about 15 min.
  11.1 What do you think of this drug checking process?
    - Any aspects you like/do not like about the process?
    - Anything you find particularly helpful/unhelpful?
    - Anything you would change about/add to the process?
    - Anything missing from the process?
12. We are now going to show you some of the type of information you may receive after submitting your sample for drug checking. This includes an information sheet on the type of drug you nominated for testing and some harm reduction strategies
  – What do you think about the way this information is provided?
  – Do you find the type of information provided interesting/not interesting?
  – Do you find the type of information provided helpful/unhelpful
  – Is there anything you would change about the way this information is provided?
  – Is there anything missing or not relevant?
13. Colour reagent drug checking works by mixing a small scraping from a drug (< 0.01 g/one‐tenth of a point) with a specific liquid (determined based on the presumed class of the drug being tested). The liquid, which changes in colour after mixing with the drug, is then compared to a reference chart to determine the presence/absence of the presumed substance. The advantage of colour reagent tests is that they require only a small drug sample (< 10 mg, which is one tenth of a point); they are quick (results generally available in < 10 min); and they are relatively inexpensive. However, colour reagent drug checking does not work well for detecting multiple drug mixtures (e.g., MDMA with methamphetamine) and the presence of adulterants (i.e., what the drug has been cut/mixed with, e.g., caffeine, levamisole [a medication used to treat parasitic worm infections in animals]) and it cannot establish the purity of a substance. This is a problem as certain drug mixtures and adulterants may be associated with increased risk of adverse outcomes.
  13.1 What do you think of the use of these colour‐reagent tests in drug checking services?
  13.2 What would you think/do with the drug if a colour‐reagent test showed that the drug you tested did not contain the substance you thought it did? (e.g., you thought you were testing methamphetamine, but the colour reagent test indicates the absence of methamphetamine in the sample)
  13.3 What would you think/do with the drug if a colour‐reagent test showed that the drug you tested did contain the substance you thought it did? (e.g., you thought you were testing methamphetamine and the colour reagent test indicates the presence of methamphetamine)
14. Other drug checking tests (e.g., spectrometry methods) can provide more detailed information than colour‐reagent tests, including information on drug purity, and the number and quantity (i.e., percentage) of drugs and adulterants (i.e., other substances cut/mixed with the drug) present in a drug sample. However, these methods are much more expensive than colour reagent tests and take a longer time to process as this method requires specialised equipment and a person with expertise in the testing method to analyse the drug sample. So, the wait time for results using this method can range from 1 h up to 3 weeks, depending on how common the drugs being tested and the mixtures within the sample are. These tests may also require a larger sample of the drug to be donated for testing (e.g., a full tablet/capsule; or 1 point).
  14.1 What do you think of the use of these more detailed tests in drug checking services?
  14.2 What would you think/do with the drug if a test showed that the drug you tested contained the substance you thought it did, but was much purer/potent than you thought it was?
  14.3 What would you think/do with the drug if a test showed that the drug you tested contained other substances in addition to the substance you thought it contained?
    - Other illicit drugs?
    - New psychoactive substances (e.g., bath salts/cathinones)?
    - Adulterants such as caffeine, paracetamol (Panadol) or Levamisole (a medication used to treat parasitic worm infections in animals)
