# Supplementary material for: ‘It'd Be Nice to Know if You're About to Have Drain Cleaner’: A Qualitative Study of Preferred Drug Checking Service Features to Inform the Design of CheQpoint, Queensland's First Fixed‐Site Drug Checking Service
Source: Drug Alcohol Rev. 2025 Jun 30;44(6):1772–85. doi: 10.1111/dar.70003 (PMC12405825; doi:10.1111/dar.70003)
Supplement: Supplementary file 1 — Table S1. Consolidated criteria for reporting qualitative studies (COREQ): 32‐item checklist. [file DAR-44-1772-s001.docx]

# Table S1. Consolidated criteria for reporting qualitative studies (COREQ): 32-item checklist

| **Item number** | **Topic** | **Guide questions/description** | **Reported Page/s** |
| --- | --- | --- | --- |
| **Domain 1: Research team and reflexivity** | | |  |
| *Personal characteristics* | | |  |
| 1 | Interviewer/ facilitator | Which author/s conducted the interview or focus group?  **BCD, MC and GN conducted the interviews.** | 5-6 |
| 2 | Credentials | What were the researchers’ credentials? E.g. PhD, MD  **CP – BSocSci (Hons), PhD**  **NP – PhD**  **BCD – MPsych (Clin), PhD**  **MB – BSc(Psych)(Hons), PhD**  **CF – Bachelor of Social Work, Master of Health Studies (Addiction Studies)**  **DS – PhD**  **LH – PhD (Clin Psych)** | 6 |
| 3 | Occupation | What was their occupation at the time of the study?  **CP – Senior Research Fellow**  **NP – Research Fellow**  **BCD – Research Assistant**  **MB - Senior Research Fellow**  **CF – CEO of The Loop Australia**  **DS – Senior Research Fellow**  **LH – Professor** | 6 |
| 4 | Gender | Was the researcher male or female?  **One interviewer was male (BCD); the other two were female (MC, GN)** | 6 |
| 5 | Experience and training | What experience or training did the researcher have?  **All interviewers had a bachelor (honours) degree in psychology. BCD also had a PhD in Psychology. All interviewers were completing a postgraduate degree in clinical psychology, had experience working with clients, and were trained in qualitative interviewing.** | 6 |
| *Relationship with participants* | | |  |
| 6 | Relationship established | Was a relationship established prior to study commencement?  **No members of the research team had established relationships with any study participants** | 6 |
| 7 | Participant knowledge of the interviewer | What did the participants know about the researcher? e.g. personal goals, reasons for doing the research  **The consent form outlined that the study aimed to better understand the potential barriers and facilitators to accessing drug checking services, perceptions of drug checking services, and which features of a drug checking service were most appealing or useful for potential service clients.** | N/A |
| 8 | Interviewer characteristics | What characteristics were reported about the interviewer/ facilitator? e.g. Bias, assumptions, reasons and interests in the research topic  **As interviews were conducted in person or via Zoom, participants were able to see researchers. Each researcher commenced each interview by introducing themselves and briefly explaining their background and current position.**  **The consent form outlined that the study aimed to better understand the potential barriers and facilitators to accessing drug checking services, perceptions of drug checking services, and which features of** **a drug checking service were most appealing or useful for potential service clients.** | 6 |
| **Domain 2: Study design** | | |  |
| *Theoretical framework* | | |  |
| 9 | Methodological orientation and theory | What methodological orientation was stated to underpin the study? e.g. grounded theory, discourse analysis, ethnography, phenomenology, content analysis  **Iterative categorisation.** | 6 |
| *Participant selection* | | |  |
| 10 | Sampling | How were participants selected? e.g. purposive, convenience, consecutive, snowball  **Convenience sample** | 5 |
| 11 | Method of approach | How were participants approached? e.g. face-to-face, telephone, mail, email  **Participants were approach one of three ways: (1) Email; (2) in-person via Lives Lived Well inpatient residential alcohol and other drug treatment programs; and (3) in-person at QuIHN.** | 5 |
| 12 | Sample size | How many participants were in the study?  **N=56** | 7 |
| 13 | Non-participation | How many people refused to participate or dropped out? Reasons?  **Participants self-selected to participate in the online survey and interview. No participants dropped out of the study.** | N/A |
| *Setting* | | |  |
| 14 | Setting of data collection | Where was the data collected? e.g. home, clinic, workplace  **Interviews were conducted in-person with clients recruited via LLW and QuIHN, and via Zoom with clients recruited online.** | 5-6 |
| 15 | Presence of non-participants | Was anyone else present besides the participants and researchers?  **No.** | N/A |
| 16 | Description of sample | What are the important characteristics of the sample? e.g. demographic data, date  **The final sample comprised N=56 participants (18 LLW; 20 QuIHN, 18 Online). 32 identified as male and mean age was 34.9 years (SD = 9.7). Participants’ socio-demographic characteristics are reported in Table 1.** | 24 |
| *Data collection* | | |  |
| 17 | Interview guide | Were questions, prompts, guides provided by the authors? Was it pilot tested?  **A semi-structured qualitative interview with prompts was used as an interview guide. The interview was not pilot tested.** | 6 and 28-32 |
| 18 | Repeat interviews | Were repeat interviews carried out? If yes, how many?  **No.** | N/A |
| 19 | Audio/visual recording | Did the research use audio or visual recording to collect the data?  **Yes**. **Interviews were audio recorded with participant permission.** | 6 |
| 20 | Field notes | Were field notes made during and/or after the interview or focus group?  **No.** | N/A |
| 21 | Duration | What was the duration of the interviews or focus group?  **Interviews ranged from 21 to 51 minutes in duration (Mean= 34 minutes; SD=9 minutes).** | 6 |
| 22 | Data saturation | Was data saturation discussed?  **Yes. Interviews were conducted until data saturation was obtained, as determined by consensus between the interviewers and research team.** | N/A |
| 23 | Transcripts returned | Were transcripts returned to participants for comment and/or correction?  **No.** | N/A |
| **Domain 3: Analysis and findings** | | |  |
| *Data analysis* | | |  |
| 24 | Number of data coders | How many data coders coded the data?  **Two. The primary coder (BCD) coded all interviews and the secondary coder (SG) coded a subsample of 6 interviews (10%). NP then examined inter-coder reliability and resolved any coding discrepancies between BCD and SG.** | 6 |
| 25 | Description of the coding tree | Did authors provide a description of the coding tree?  **No.** | N/A |
| 26 | Derivation of themes | Were themes identified in advance or derived from the data?  **Interviews were coded using the semi-structured interview protocol and research team knowledge of the area as a guide (deductive approach), supplemented by an inductive approach whereby the coder also allowed the creation of ideas that came-up organically.** | 6 |
| 27 | Software | What software, if applicable, was used to manage the data?  **NVivo (Release 1.7).** | 6 |
| 28 | Participant checking | Did participants provide feedback on the findings?  **No.** | N/A |
| *Reporting* | | |  |
| 29 | Quotations presented | Were participant quotations presented to illustrate the themes / findings? Was each quotation identified? e.g. participant number  **Yes. Quotations are identified by gender, age, and place of recruitment (i.e., online, LLW, QuIHN).** | 7-16 |
| 30 | Data and findings consistent | Was there consistency between the data presented and the findings?  **Yes.** | N/A |
| 31 | Clarity of major themes | Were major themes clearly presented in the findings?  **Yes.** | 7 |
| 32 | Clarity of minor themes | Is there a description of diverse cases or discussion of minor themes?  **Yes, there is discussion of diverging ideas among study participants, where applicable.** | 7-16 |
